# Basalt-Based Composite with Reduced Graphene Oxide (rGO)—Preliminary Study on Anti-Cut Properties

**DOI:** 10.3390/ma18245513

**Published:** 2025-12-08

**Authors:** Agnieszka Cichocka, Iwona Frydrych, Piotr Zawadzki, Łukasz Kaczmarek, Emilia Irzmańska, Paulina Kropidłowska

**Affiliations:** 1 Institute of Architecture of Textiles, Lodz University of Technology, Zeromskiego 116 Str., 90-543 Lodz, Poland; iwona.frydrych@p.lodz.pl; 2 Institute of Materials Science and Engineering, Lodz University of Technology, Stefanowskiego 1/15, 90-924 Lodz, Poland; piotr.zawadzki@p.lodz.pl (P.Z.); lukasz.kaczmarek@p.lodz.pl (Ł.K.); 3 Department of Personal Protective Equipment, Central Institute for Labour Protection—National Research Institute, Wierzbowa 48, 90-133 Lodz, Poland; emirz@ciop.lodz.pl (E.I.); pakro@ciop.lodz.pl (P.K.)

**Keywords:** basalt-based composite, rGO, anti-cut properties, protective clothing, protective materials, PPE

## Abstract

This study investigates the anti-cut properties of a composite based on basalt fabrics with varied structural characteristics, including weave and thread density, enhanced with reduced graphene oxide (rGO). The primary aim is to evaluate the potential of integrating rGO into a basalt matrix to improve its resistance to cutting and mechanical damage. The results indicate that adding rGO significantly increases the cutting resistance of the composite. Molecular simulations demonstrate that the composite, which combines a cross-linked LG 700 resin, rGO, and basalt, is one of the most thermodynamically stable configurations due to strong electrostatic interactions between its components. These interactions and the formation of physical bonds at the interfaces stiffen the material, while also allowing for a unique crack-toughening effect. This resilience, which enables the reformation of physical interactions after stress, directly contributes to the composite’s enhanced resistance to catastrophic failure and its observed performance in cutting tests. These findings suggest that basalt–resin with rGO composites hold great potential for applications requiring high mechanical strength and durability, such as protective clothing (e.g., gloves) and anti-vandalism materials. The study concludes that the developed composite represents a promising advancement for materials exposed to cutting forces.

## 1. Introduction

The demand for advanced protective materials with enhanced resistance to mechanical damage, especially cutting and tearing, has significantly increased across various sectors, including industry, public infrastructure, and defense. Anti-cut and anti-vandalism solutions are crucial for ensuring the safety of personnel and extending the durability of equipment and infrastructure exposed to harsh operational or environmental conditions. Irzmańska et al. [1] conducted studies on fire-retardant hybrid composites modified with inorganic and organic fillers. Their findings indicate that the presence and quantity of glass reinforcement had a significant effect on the impact strength of the fabricated composites. This illustrates the importance of material composition in achieving superior mechanical resistance. Moreover, a lower impact strength was observed for the composites, which had powder fillers causing the formation of micro-voids in the structures and thus led to a weakening in their strength properties. In another work, Irzmańska et al. [2] modified standardized procedures intended for testing personal protective equipment, including impact, cut resistance, and flame resistance, to assess the properties of the anti-vandal hybrid composites.

One of the most prominent application areas for such materials is protective clothing, including gloves. Industrial workers in metalworking, construction, automotive manufacturing, and glass processing often face the risk of injury from sharp tools, sheet metal, or broken glass. In these contexts, garments made with cut-resistant fabrics are essential for improving workplace safety without compromising comfort and flexibility. Similarly, anti-vandal materials are employed in transportation systems, public seating, protective casings for electronics, and architectural surfaces to reduce damage caused by intentional acts of destruction or wear.

A promising direction in the development of protective textiles is the use of basalt fibers, known for their excellent thermal resistance, mechanical strength, and chemical stability. The need to develop and improve protective clothing incorporating such fibers has been highlighted in several studies, which present successful examples of innovative material solutions. Notable contributions include works by Gilewicz et al. on underwear for foundry workers [3] and Frydrych et al. on comparative thermal insulation analyses [4], as well as studies on the surface modification of basalt fabrics for high-temperature protection using magnetron sputtering [5].

### 1.1. Basalt Fibers

Among the materials that meet strict requirements for mechanical, thermal, and chemical resistance, basalt fibers stand out due to their excellent balance of functional properties. Derived from dense and hard volcanic rocks, they are known for their exceptional durability and high-temperature resistance. Their production uses a raw material with a precisely defined chemical composition, enabling the manufacture of continuous fibers with diameters ranging from 9 to 24 µm and controlled properties.

Due to its wear resistance, basalt was primarily used in foundry applications, such as the production of architectural slabs or inserts in steel pipes. Today, basalt fibers are finding increasingly wide applications thanks to their low moisture absorption, low thermal conductivity, minimum elongation at break, and high durability—they do not undergo erosion or corrosion [6,7,8,9,10].

Another advantage of basalt fibers is their lower cost compared to glass and carbon fibers, while still providing comparable or even superior levels of protection [11,12]. They also exhibit excellent resistance to acids and chemical inertness—they do not react with air or water, are non-flammable, and are explosion-resistant. Ref. [9] also highlights basalt’s potential for improving occupational safety and air quality. Importantly, the fiberization process for basalt is more environmentally friendly than glass fibers.

A comparative analysis of the properties of basalt fibers and traditionally used E-glass fibers is presented in Table 1. Basalt fibers demonstrate superior mechanical and thermal properties compared to E-glass fibers.

They exhibit a higher tensile strength and a greater Young’s modulus, indicating better stiffness and overall strength than E-glass fibers. Basalt fibers also have a much wider operating temperature range and higher short-term heat resistance—up to about 200 °C—than E-glass fibers. Their melting point is significantly higher, reaching up to 1460 °C, what confirms their excellent thermal stability. However, basalt fibers show lower elongation at break, which means they are less flexible and more brittle than glass fibers. This brittleness is one of the reasons why basalt fabrics are often reinforced with resin to improve their mechanical performance. In terms of thermal performance, basalt fibers provide better thermal insulation and maintain their properties over a much wider operating temperature range (from −260 °C to +700 °C) compared to E-glass fibers (−60 °C to +380 °C). This superior thermal stability makes basalt fibers more suitable for use in protective clothing designed for high- and low-temperature environments, where reliable insulation and resistance to heat are essential.

### 1.2. Properties of Graphene–rGO—Strength, Stiffness, and Chemical Resistance

The main difference between graphene and reduced graphene oxide (rGO) lies in their chemical structure and purity. Graphene is a pure, two-dimensional structure composed solely of carbon atoms, forming a honeycomb structure. rGO is formed by reducing graphene oxide (GO), meaning that although most of the oxygen groups have been removed, it still contains structural defects and trace amounts of oxygen and other elements. Since rGO has properties similar to those of graphene but is a slightly worse material due to defects, the basic information about graphene is presented.

Graphene, a single-atom-thick layer of carbon arranged in a honeycomb lattice, is one of the most promising materials of the 21st century due to its unique physical, chemical, and mechanical properties.

Graphene exhibits an exceptionally high tensile strength—estimated to be about 200 times stronger than steel of the same thickness. Its tensile strength reaches approximately 130 GPa, making it the strongest known material at the nanoscale. This strength makes graphene an ideal candidate for reinforcing composite materials [12].

Characterized by a very high stiffness, graphene has a Young’s modulus of about 1 TPa (terapascal). This means the material is highly resistant to deformation under external forces, making it suitable for applications requiring high stability and resistance to mechanical strain [13].

In addition, graphene exhibits excellent chemical resistance—it is chemically inert to most organic and inorganic compounds, does not corrode, and is insoluble in water. Thanks to its high chemical stability, it can be used in harsh environments exposed to acids, bases, or UV radiation [14].

Moreover, graphene conducts heat and electricity better than copper, making it attractive for electronic applications. Additionally, after chemical modification it can be used for thermal insulation applications. In composites, the addition of graphene can significantly enhance the mechanical and barrier properties of traditional materials, including basalt and glass fibers [15].

### 1.3. Research Examples of Comparable Composites

A literature review indicates that laminated composites based on epoxy resin reinforced with basalt fibers exhibit favorable mechanical properties often comparable to or exceeding those of glass fiber composites. These composites are commonly produced by manual lay-up with pressure curing. Microscopic analysis after mechanical testing confirms good structural integrity, low porosity, and limited fiber delamination. Due to their low cost and safety, basalt-based materials present a promising alternative to traditional glass fiber composites [16].

Another publication on composites utilizing basalt and graphene compared the mechanical properties of pure basalt composite pipes with those modified by graphene nanoplatelets. Adding graphene significantly improved the stiffness and ductility of the material. Various mechanical tests were conducted, including tensile, impact, hardness, bending, and dynamic vibration tests, revealing complex frequency-dependent mechanical behavior. These results confirm the potential of basalt–graphene epoxy composites for applications requiring enhanced mechanical performance [17].

Graphene-coated fiber-reinforced polymer composites have emerged as promising materials for a wide range of applications [18,19,20]. These include their use as sensors for real-time structural health monitoring and non-destructive inspection, as well as in fracture mechanics studies. The piezoresistive properties, high selectivity, and fast response time of graphene make it well-suited for use in strain, gas, and electrochemical sensors [21,22,23].

Although basalt fibers and graphene additives have attracted increasing interest, previous research has primarily focused on their use in the construction and automotive sectors. There is a lack of studies analyzing their synergistic use in composites intended for protective clothing, particularly in terms of cut and mechanical damage resistance. Here, we try to fill this research gap.

Basalt-based composites—especially those enhanced with functional additives such as rGO—represent a promising class of materials for anti-cut applications, including industrial and military applications. Composites based on basalt fabrics and graphene embedded in epoxy resin are advanced materials that combine the unique properties of three components: basalt fabrics, epoxy resin, and rGO, offering a range of benefits in various engineering and industrial applications.

The goal is to better understand the interactions at the molecular level and translate them into macroscale properties relevant to protective applications.

## 2. Materials and Methods

### 2.1. Materials Tested

Table 2 presents four samples of fabrics made entirely from basalt fiber yarn which were subjected to physical and structural property testing. Each sample differs in terms of mass per square meter, thickness, weave, and warp/weft thread density, which differences affect their properties.

The first sample, woven in a 3/1 S warp-faced twill, has the highest mass per square meter (805 g/m^2^) and thickness (0.91 mm). It features good drapability, a pronounced surface texture, and a visible structure. Yarn slippage is lower compared to the other samples, resulting in better dimensional stability.

The second sample has a lower areal density (385 g/m^2^) and thickness (0.49 mm), woven in plain weave. Like the first, it offers good drapability and a distinct surface texture, but visible gaps and high yarn slippage in both the warp and weft directions contribute to greater fabric flexibility.

The third sample, with the lowest areal density (286 g/m^2^) and thickness (0.31 mm), is also plain-woven. It has a dense structure, is less flexible, and feels stiff to the touch, with some yarn slippage observed in both the warp and weft directions.

The fourth sample is made from aluminized basalt fiber yarn, with an areal density of 228 g/m^2^ and a thickness of 0.29 mm, also in plain weave. It shows good drapability, a distinct surface texture, no visible gaps, and no yarn slippage in either the warp or weft directions, indicating high structural stability.

These selected basalt fiber fabric samples subsequently underwent cut resistance testing (according to the relevant standard), both in their original form and after being treated with a graphene-enhanced resin.

Table 3 compares the mechanical properties of basalt fibers and E-glass fibers (data from [6]). Basalt fibers exhibit a higher tensile strength, stress resistance, and modulus of elasticity than E-glass fibers.

The differences in physico-thermal properties arise from variations in the chemical composition and the percentage content of the individual components of the fibers, as presented in Table 4 (adapted from [8]).

They are also influenced by differences in fiber diameter, as shown in Table 1 (data from [26]).

### 2.2. Cut Resistance Evaluation Methods

Three standardized cut resistance tests were conducted to assess the mechanical protective performance of the basalt fabric samples—both in their raw form and after graphene–epoxy impregnation. Each method reflects a different type of mechanical hazard relevant to protective glove applications.

A. Knife Impact Test—EN 1082-3:2000—protective clothing—gloves and arm guards protecting against cuts and stabs by hand knives. Part 3: Impact cut test for fabric, leather, and other materials [27].

This test evaluates the resistance to cutting due to a knife impact. A specially designed blade is dropped onto the material with a controlled impact energy of 2.45 joules, simulating a dynamic cutting threat such as a slash or stab. The resulting cut length is measured and used as an indicator of material resistance. This method is widely used for assessing protective gloves and clothing designed to reduce the risk of injury from sharp objects. It was chosen for this study due to its relevance to real-world occupational hazards.

B. Knife Penetration Test—EN ISO 13998:2003—protective clothing—aprons, trousers, and vests protecting against cuts and stabs by hand knives [28].

This method also involves a knife strike at 2.45 J of impact energy but places stronger emphasis on evaluating penetration resistance, simulating stab threats in industrial or security environments. The sample is mounted on a compliant base, and the cut length is again recorded. Importantly, this standard introduces a classification scale, assigning protection levels based on the length of the resulting cut, shorter cuts indicating better protection. This method complements the first test and helps identify materials with potential application in high-risk sectors, including food processing and defense.

C. Blade Cut Resistance—EN ISO 13997:2023 (TDM Test)—protective clothing—mechanical properties—determination of resistance to cutting by sharp objects [29].

This method assesses the resistance of materials to being cut through by a sharp blade under a constant force rather than an impact. A straight blade is drawn across the fabric with an applied force of 150 N (in this study), and the length of the cut required to breach the material is measured. The test provides a quantitative measure of cut-through resistance and is especially useful for comparing textiles and composites with high mechanical integrity. The results indicate exceptionally high protection performance when no visible penetration is observed. This test was included to evaluate steady-force threats such as slicing or sawing motions.

These three complementary methods provide a comprehensive evaluation of the protective potential of basalt–graphene composites, reflecting different real-world threats: dynamic slashing, stabbing, and constant-pressure cutting. Their combined use offers a robust foundation for assessing suitability in technical and protective textile applications.

### 2.3. Composite Preparation: Vacuum-Assisted Resin Infusion with Graphene-Modified Epoxy System

The composite material was fabricated using the vacuum-assisted resin infusion technique (VARI), in which basalt fabrics were impregnated with an epoxy resin system modified with reduced graphene oxide (rGO). Detailed information about rGO, which is a modified version of graphene, is presented by Kaczmarek et al. [30].

Previous preliminary work, specifically master’s thesis projects [31,32] focusing on epoxy resins with graphene powder, although they considered different shares of rGO, provided critical insight: they demonstrated the potential to achieve superior mechanical properties. Consequently, leveraging the foundational knowledge gained from these prior student investigations, the focus of the current publication is analyzing the incorporation of resin with rGO powder, omitting the step of a research trial with the resin only. Prior to the preparation of the mixture, the structural and physical properties of the rGO powder were thoroughly characterized to ensure quality control.

**Figure 1:**
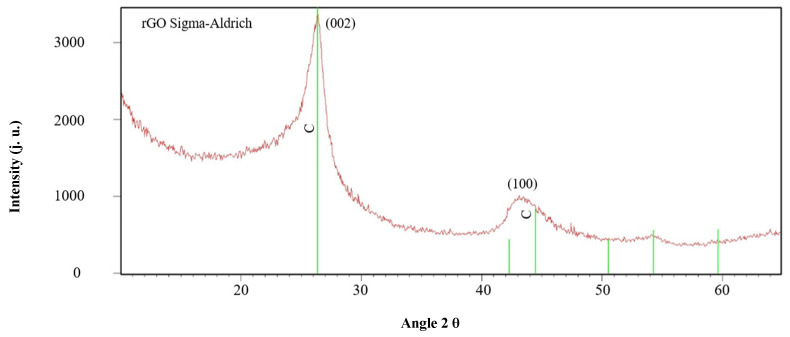
XRD spectrum for rGO material (Sigma-Aldrich, Stainheim, Germany).

The results obtained via X-ray diffraction (XRD) are summarized in Table 5 and presented graphically in Figure 1.

**Figure 2:**
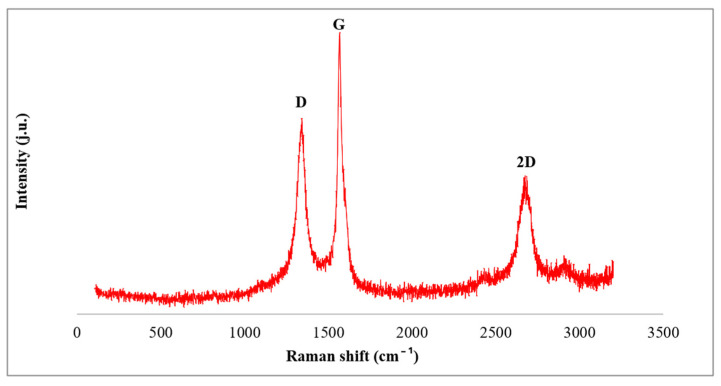
Raman spectra for rGO (Sigma -Aldrich, Stainheim, Germany).

The characteristic Raman bands and structural coefficients are provided in Table 6 and presented graphically in Figure 2.

Our approach was unable to employ ultrasonic mixing due to the high temperatures generated within the rGO–resin mixture, which resulted in uncontrolled cross-linking (curing). Therefore, mechanical mixing at ambient temperature at 1500 rpm was used, and additional thinning of the inlet channel was used during the infusion stage to generate shear forces that caused additional graphene dispersion.

To prepare the resin mixture, 0.1 wt% of rGO (based on the total resin mass) was first dispersed into the HG 700 hardener through mechanical stirring for 3 min. This dispersion was then added to the LG 700 epoxy resin and mixed intensively for another 2 min, yielding a homogenous resin–hardener–rGO suspension.

The final mixture was degassed under vacuum for 1.5 min to eliminate air bubbles and trapped gases. Once degassed, the modified resin was used to impregnate basalt fiber mats via resin infusion under vacuum pressure, ensuring thorough wetting and uniform matrix distribution throughout the fiber structure.

This method facilitated the controlled incorporation of rGO into the epoxy matrix, thereby enhancing the mechanical and functional performance of the resulting basalt-based composites.

## 3. Results and Discussion

### 3.1. Composite Preparation

Composite samples prepared as described in Section 2.3 were subsequently tested to evaluate their mechanical performance and anti-cut properties. The experimental procedures included cutting resistance tests and standard material characterization to assess the effect of rGO addition on the basalt-based composites.

### 3.2. Cut Resistance Evaluation

Basalt fabric samples impregnated with uncoated basalt fabrics (without resin or rGO) and graphene-containing resin (which changed color) were subjected to impact resistance tests. The results for basalt fabric without impregnation with graphene-containing resin are presented in Table 7.

According to the PN-EN ISO 13998:2003 standard, cut resistance by knife impact is assessed based on the length of the cut after a blade impact with an energy of 2.45 J. A shorter cut length indicates better material resistance. In our case, a cut length of around 55 mm yielded the following results:

Samples 1 and 3—no visible cut (0 mm)—indicate very high cut resistance according to the standard; the material effectively prevents penetration. Sample 4—a slight cut—indicates moderate resistance. Sample 2 (assumed to be the one with a relatively large cut) exhibited a larger cut length, suggesting lower cut resistance.

As per the standard, Samples 1 and 3 meet high cut resistance requirements, demonstrating excellent durability against knife impact. Samples 2 and 4 show lower resistance and may require further optimization for protective applications. The absence or minimal length of cuts confirms the anti-cut protection offered by the material.

Cut resistance against sharp objects according to EN ISO 13997:2023 (all samples tested with a force of 150 N): Samples 2 and 3 showed no visible cut, and Samples 1 and 4 exhibited slight visible cuts.

This indicates that Samples 2 and 3 provide higher protection against cutting under the applied force, while Samples 1 and 4 show some vulnerability but still limited damage.

The basalt fiber fabrics tested without resin or graphene showed excellent cut resistance, with some samples effectively preventing all penetration under dynamic knife impact and static force. Samples 1 and 3 demonstrated very high resistance, while Samples 2 and 4 showed slightly lower but still notable protective properties. These results highlight the material’s inherent robustness and suitability for demanding protective applications, confirming its potential for use in protective clothing and technical textiles even before applying additional reinforcement or treatment.

The results for basalt fabrics with impregnation with rGO-containing resin are presented in Table 8.

The results of the blade impact cut resistance test (EN 1082-3:2000, energy: 2.45 J) showed varying levels of protection among the samples:

Sample 3 shows the highest resistance, with no cut observed beyond 55 mm, indicating excellent cut protection. Samples 2 and 4 have moderate resistance, with cut lengths of 41.5 mm and 32.5 mm, respectively. Sample 1 has the lowest resistance, with a cut length of 19 mm, suggesting lower protective performance against blade impact.

Cut Resistance to Sharp Objects (EN ISO 13997:2023, force: 150 N):

Sample 3 again performed best, with a cut length of 60.6 mm, confirming its superior cut resistance. Samples 2 and 4 showed similar results (56.4 mm and 59.7 mm), indicating moderate protective capabilities. Sample 1 could not be measured using the TDM device due to its small size and stiffness, which highlights the limitations of this sample in such testing. These limitations suggest that its unique structure is not well-suited for this specific testing method, which is a key factor to consider in its application.

Overall, Sample 3 demonstrated the best protective properties against both blade impact and sharp object cutting. Sample 1 showed the lowest resistance and exhibited mechanical limitations that prevented a complete assessment under all conditions. These results underscore the critical role that fabric structure and parameters play in effective cut protection performance.

### 3.3. Molecular Simulations

To investigate the physicochemical interactions at the atomic scale and their relationship to macroscopic mechanical properties, quantum chemical calculations were performed using the semi-empirical PM6 method. These analyses were conducted using Fujitsu Scigress 2.9 software and involved constructing and optimizing 3D molecular models for cross-linked LG 700 epoxy resin, graphene flakes, and basalt fibers.

Molecular geometries were fully optimized in vacuum, with convergence criteria set to a gradient norm of 0.05 kcal/mol/Å. The systems contained up to 357 primarily atoms (C, H, and O), and no periodic boundary conditions were applied. The electron density distribution of the macromolecules was analyzed to evaluate electrostatic interactions. Changes at the phase boundaries between epoxy resin, basalt, and graphene were studied, considering both electrostatic effects. Analyses were also conducted for conditions in which the QGDs were isolated from their surroundings (without additional external interactions). Due to the presence of primarily three types of atoms (O, H, and C), a semi-empirical method was used, which enabled relatively fast calculations.

#### 3.3.1. Stage 1—Modeling of Pure Components

Initially, individual components were modeled: a cross-linked molecule of LG 700 resin, a fragment of basalt fiber, and a graphene structure. According to Table 8, the calculated total energies of these systems were as follows: LG 700 epoxy resin: −393 kcal/mol; basalt fiber: −4279 kcal/mol; graphene: +225 kcal/mol.

**Figure 3:**
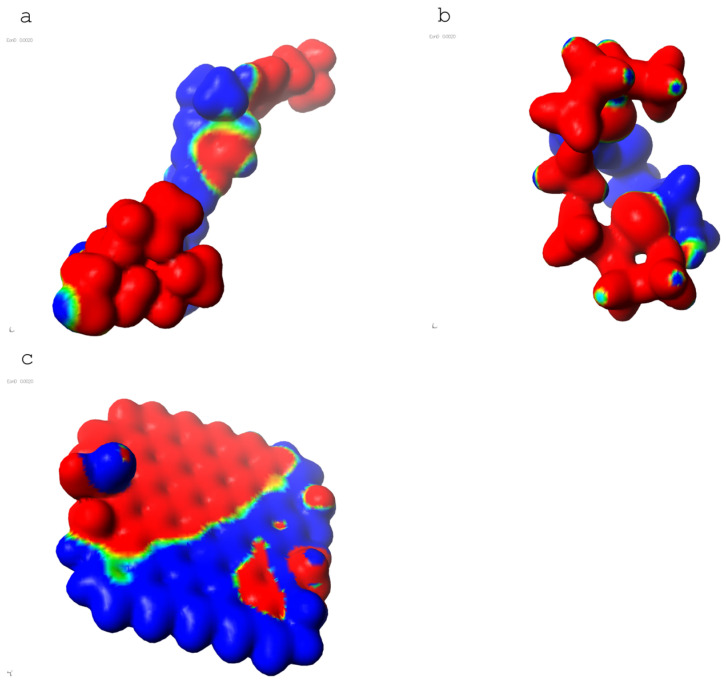
Electron distribution in the analyzed (**a**) cross-linked LG 700 resin molecule, (**b**) basalt fiber fragment, and (**c**) graphene structure.

Additionally, electron density distributions for each component were analyzed (see Figure 3).

#### 3.3.2. Stage 2—Pairwise Interactions

Next, molecular interactions were simulated for the following phase boundaries:

(a) Cross-linked LG 700 basalt resin–basalt;

(b) Basalt–rGO;

(c) Cross-linked LG 700 resin–rGO.

The respective calculated interaction energies were as follows:

(a) −4677 kcal/mol; (b) −4055 kcal/mol; (c) −178 kcal/mol (Table 8).

Electron distribution mapping was also performed for these systems.

#### 3.3.3. Stage 3—Ternary System Modeling

Finally, the whole composite system—cross-linked LG 700 resin–rGO–basalt—was modeled. This system exhibited an interaction energy of −4439 kcal/mol (Table 9). Electron density distributions were also analyzed for this complex macromolecular structure (see Figure 3).

### 3.4. Summary

The results indicate that the composite structure combining cross-linked LG 700 resin, rGO, and basalt is one of the most thermodynamically stable among the analyzed configurations. This is attributed to electrostatic interactions at each interface:

(a) Resin–basalt;

(b) Basalt–rGO;

(c) Resin–rGO.

**Figure 4:**
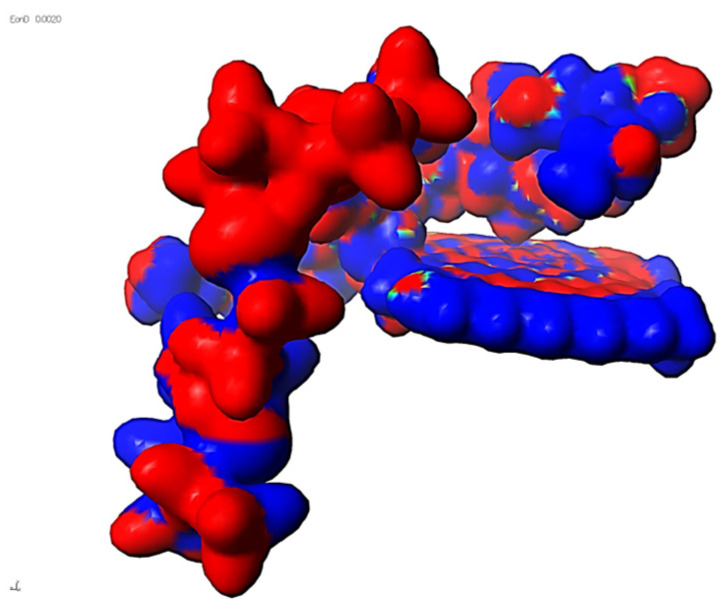
Electron distribution at the triplet interface: cross-linked LG 700 resin molecule–rGO–basalt.

**Figure 5:**
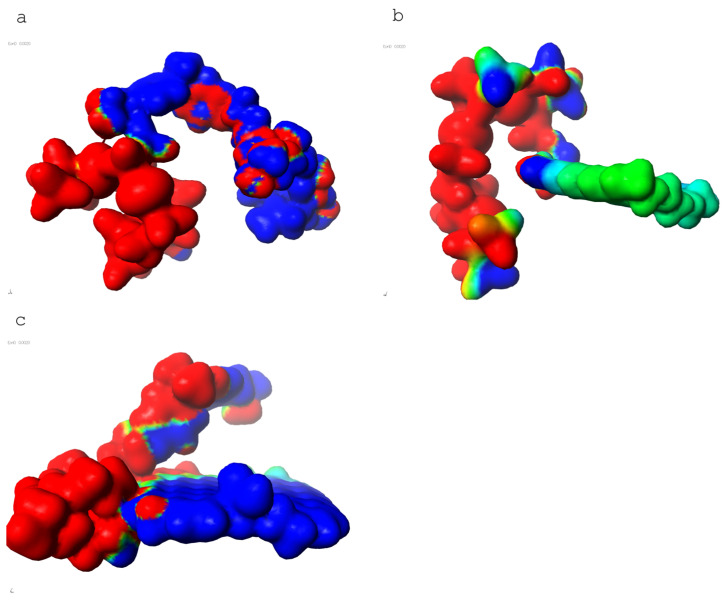
Electron distribution at the interfaces of the pairs: (**a**) cross-linked LG 700 resin molecule–basalt; (**b**) basalt–rGO; and (**c**) cross-linked LG 700 resin molecule–rGO.

The results’ analysis proves that the composite consisting of a cross-linked LG 700 resin molecule–rGO–basalt is one of the most thermodynamically stable systems among those analyzed (Figure 4). This is because the electrostatic interactions are formed as a result of the presence of elements with different polarities in their molecules: C, O, N, and H (Figure 5a–c, respectively).

This leads to a variable electron distribution across the macromolecule, resulting in the formation of local dipoles that significantly contribute to system stabilization and energy reduction. Additionally, functional groups may graft at the interfaces of the analyzed systems, for instance, through the formation of chemical bonds. This phenomenon directly influences the mechanical properties of the resulting composite by both spatially stiffening its macromolecules (through new chemical bonds at the phase boundaries and the creation of local dipole interactions) and enabling a potential crack-toughening effect through the formation of physical interactions.

Particularly in the latter case, an external force field that breaks the physical interactions and causes mutual displacement of molecules does not necessarily lead to cracking. This is because breaking the physical interactions between macromolecules and subsequent displacement can be followed by the reformation of physical interactions in a different spatial configuration. This unique resilience, which prevents catastrophic failure, has a significant impact on the observed cut resistance test results.

## 4. Conclusions

Experimental tests and computer simulations have confirmed that the application of a basalt fabric-reinforced epoxy composite with the addition of 0.1 wt% (rGO) significantly improves the material’s mechanical properties, particularly its cut resistance.

According to knife impact tests (according to EN ISO 13998:2003) and sharp object cut resistance tests (EN ISO 13997:2023), samples containing graphene exhibited noticeably greater structural stability and lower susceptibility to damage compared to uncoated samples.

Electron distribution simulations demonstrated the formation of local dipoles and strong electrostatic interactions at the phase boundaries between the composite components (resin, rGO, and basalt). These interactions were found to enhance the system’s thermodynamic stability and contribute to its increased resistance to external mechanical loads. Microscopic observations revealed no visible cuts or only minimal cut marks, especially in the graphene-modified samples, confirming the effectiveness of the proposed material solution.

Future work will optimize the type, concentration, and dispersion of graphene additives, as well as explore alternative resins and impregnation methods, to improve both mechanical performance and wearability in protective textiles.

## Figures and Tables

**Table 1:** Comparative summary of the mechanical and thermal properties of basalt fibers and E-glass fibers.

Parameter	Basalt Fiber	Glass Fiber (Type E)
Fiber diameter, µm	7–22	5–20
Density, g/cm^3^	2.65	2.60
Tensile strength, MPa	4150–4800	3450
Young’s modulus, GPa	100–110	76
Elongation at break, %	3.30	4.76
Operating temperature range, °C	−260 to +700	−60 to +380
Short-term maximum heat resistance, °C	+750	+550
Melting point, °C	+1050 to +1460	+730 to +1000
Thermal insulation (conductivity), W/m^2^·K	0.031–0.038	0.034–0.040

**Table 2:** Physical and structural characteristics of basalt fiber fabrics used in the study.

No.	Fabric Description	Areal Density (g/m^2^)	Thickness (mm)	Weave	Thread Density (Threads/dm) Warp g_0_/ Weft g_t_	Warp Yarn Type	Weft Yarn Type	Other External Features
1	Fabric made of 100% basalt fiber yarn 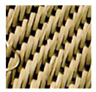	805	0.91	Warp-faced twill 3/1 S	g_0_ = 40/ g_t_ = 40	Continuous filament yarn	Continuous filament yarn	Good drapability. Pronounced surface texture. Visible gaps in the structure. Lower yarn slippage in both warp and weft directions.
2	Fabric made of 100% basalt fiber yarn 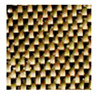	385	0.49	Plain	g_0_ = 30/ g_t_ = 30	Continuous filament yarn	Continuous filament yarn	Good drapability. Pronounced surface texture. Visible gaps in the structure. High yarn slippage In both warp and weft directions.
3	Fabric made of 100% basalt fiber yarn 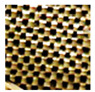	286	0.31	Plain	g_0_ = 100 g_t_ = 100	Continuous filament yarn	Continuous filament yarn	Dense fabric structure, low flexibility. Some yarn slippage in both warp and weft directions.
4	Fabric made of 100% aluminized basalt fiber yarn 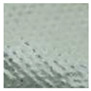 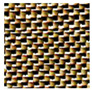	228	0.29	Plain	g_0_ = 100/ g_t_ = 80	Continuous filament yarn	Continuous filament yarn	Good drapability. Distinct surface texture. No visible gaps in the structure. No yarn slippage in either warp or weft directions.

**Table 3:** Mechanical properties of basalt fibers and E-glass fibers [6].

Mechanical Parameter	Basalt Fiber	E-Glass Fiber
Tensile Strength [24] [mN/tex]	600–730	350–500
Stress [25] [MPa]	4000–4300	3450–3800
Tensile Modulus [25] [GPa]	84–87	72–76

**Table 4:** Chemical composition of glass and basalt fibers (wt%) [8].

No.	Component [%]	E-Glass Fibers	S-Glass Fibers	C-Glass Fibers	Basalt Fibers
1	SiO_2_	52–56	65	64–68	51.56
2	Al_2_O_3_	12–16	25	3–5	18.24
3	CaO	16–25	–	11–15	5.15
4	MgO	0–5	10	2–4	1.30
5	B_2_O_3_	5–10	–	4–6	–
6	Na_2_O	0.8	0.3	7–10	6.36
7	K_2_O	–	–	–	4.50
8	TiO_2_	–	–	–	1.23
9	Fe_2_O_3_	–	–	–	4.02
10	FeO	–	–	–	2.14
11	MnO	–	–	–	0.28
12	H_2_O	–	–	–	0.46
13	P_2_O_5_	–	–	–	0.26

**Table 5:** Physical parameters characterizing graphene nanopowder obtained via X-ray diffraction (XRD) and calculations based on the Bragg (1) and Scherrer (2) equations.

2θ [°]	β [°]	H [nm]	d_002_ [nm]	*n*
25.91	3.04	2.8	0.343	~8

**Table 6:** Summary of the position and intensity of the D, G, and 2D bands and the values of the ID/IG and I2D/IG coefficients for the rGO material (Sigma-Aldrich, Stainheim, Germany).

Intensity (j. u.)			Shift (cm^−1^)			Intensity Ratio	
I_D_	I_G_	I_2D_	D	G	2D	I_D_/I_G_	I_2D_/I_G_
940.2	1412.2	549.9	1339	1567.4	2677.2	0.67	0.39

**Table 7:** Evaluation of cutting resistance of basalt fabrics with no resin.

No.	Structure of the Sample Presented via Optical Microscope OPTAtech, 10× Magnification	Knife Impact Resistance According to EN ISO 13998:2003 (Blade Impact Energy: 2.45 J)	Cut Resistance Against Sharp Objects According to EN ISO 13997:2023 (All Samples Tested with 150 N Force)
1	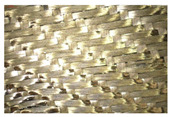	**>55 mm** 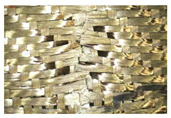	**0 mm** *No visible point of cut*
2	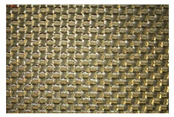	**>55 mm** 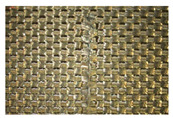	**0 mm** 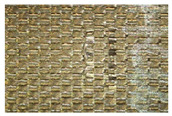
3	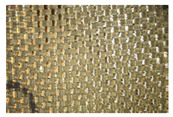	**>55 mm** *No visible point of cut*	**0 mm** *No visible point of cut*
4	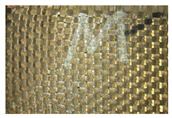	**>55 mm** 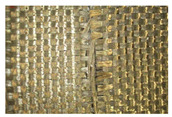	**0 mm** 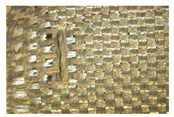

**Table 8:** Evaluation of cutting resistance of basalt fabric with impregnation with rGO-containing resin.

No.	Structure of the Sample Presented via Optical Microscope OPTAtech, 10× Magnification	Cut Resistance from Blade Impact According to EN 1082-3:2000 (Blade Impact Energy: 2.45 J)	Cut Resistance to Sharp Objects According to EN ISO 13997:2023 (All Samples Tested with 150 N Force)
1	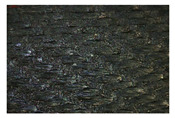	**19 mm** 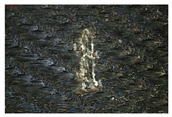	The sample was too small and stiff to allow measurement using the TDM device.
2	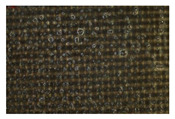	**41.5 mm** 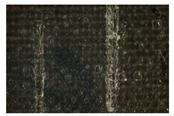	**56.4 mm** 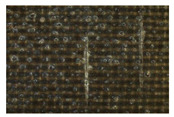
3	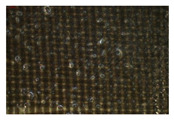	**>55 mm** 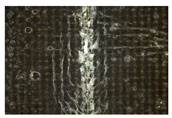	**60.6 mm** 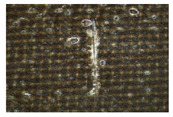
4	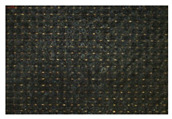	**32.5 mm** 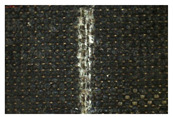	**59.7 mm** 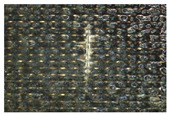

**Table 9:** Energy values of molecular systems of pure components and their mixtures.

Molecular System	System Energy Value (kcal/mol)
Cross-linked LG 700 resin	−393
rGO	+225
Basalt	−4279
Cross-linked LG 700 resin–rGO	−178
Basalt–rGO	−4055
Cross-linked LG 700 resin–basalt	−4677
Cross-linked LG 700 resin–rGO–basalt	−4439

## Data Availability

The raw data supporting the conclusions of this article will be made available by the authors on request.
